# Disease-specific factors associated with cardiovascular events in patients with Takayasu arteritis

**DOI:** 10.1186/s13075-020-02275-z

**Published:** 2020-07-31

**Authors:** Oh Chan Kwon, Jung Hwan Park, Yong-Beom Park, Min-Chan Park

**Affiliations:** Division of Rheumatology, Department of Internal Medicine, Yonsei University College of Medicine, Seoul, Korea

**Keywords:** Takayasu arteritis, Cardiovascular event, Cardiovascular risk, Methotrexate

## Abstract

**Background:**

To identify disease-specific factors associated with cardiovascular events in patients with Takayasu arteritis (TAK).

**Methods:**

Patients with TAK who fulfilled the American College of Rheumatology 1990 criteria for the classification of TAK and were followed up between 2006 and 2019 were included. Traditional cardiovascular risk factors and TAK disease-specific factors at the index date and incident cardiovascular events during the follow-up were retrospectively assessed. To estimate the risk of cardiovascular events according to TAK disease-specific factors, Cox regression analysis with adjustment for traditional cardiovascular risk factors was performed.

**Results:**

Of the total 207 patients with TAK, cardiovascular events occurred in 41 (19.8%) patients. Compared with patients who did not develop cardiovascular events, patients who developed cardiovascular events were older (38.5 ± 13.4 years vs. 43.6 ± 11.8 years, *p* = 0.028), more commonly had diabetes mellitus (6.6% vs. 19.5%, *p* = 0.029), had lower high-density lipoprotein cholesterol (57.3 ± 17.1 mg/dl vs. 51.2 ± 15.7 mg/dl, *p* = 0.040), more commonly had type V vascular involvement (33.1% vs. 63.4%, p 0.001), and less commonly received methotrexate (65.1% vs. 43.9%, *p* = 0.013). In Cox regression analysis, type V vascular involvement was significantly associated with increased risk of cardiovascular events (adjusted HR 2.852, 95% CI 1.474–5.518, *p* = 0.002), whereas the use of methotrexate was associated with reduced risk of cardiovascular events (adjusted HR 0.515, 95% CI 0.268–0.993, *p* = 0.047).

**Conclusion:**

Type V vascular involvement was associated with increased risk of cardiovascular events, while the use of methotrexate was associated with reduced risk of cardiovascular events, in patients with TAK.

## Background

Takayasu arteritis (TAK) is a chronic inflammatory disease that mainly involves the aorta and its major branches [1]. Previous studies reported higher prevalence of traditional cardiovascular risk factors in patients with TAK than in healthy controls [2–4]. Moreover, accelerated atherosclerosis has been clearly documented in TAK [5, 6], and the incidence of cardiovascular events is higher in patients with TAK than in age- and sex-matched controls [4]. Considering the relevance of cardiovascular events in patients’ morbidity and mortality, it is important to identify patients at higher risk of cardiovascular events so that appropriate prevention can be implemented.

Increased morbidity and mortality from cardiovascular events have been reported in other inflammatory rheumatic diseases such as rheumatoid arthritis (RA), ankylosing spondylitis, and systemic lupus erythematosus [7–9]. The increased risk of cardiovascular events in these diseases is only partly explained by increased prevalence of traditional cardiovascular risk factors such as age, sex, dyslipidemia, hypertension, smoking, obesity, and diabetes mellitus [10]. Systemic inflammation, in addition to traditional cardiovascular risk factors, has been suggested as the main contributor to cardiovascular events in inflammatory rheumatic diseases [10]. Indeed, there is a well-known association between chronic inflammation and atherosclerosis [11–13].

In patients with TAK, it is unclear whether TAK disease-specific factors also account for accelerated atherosclerosis and increased cardiovascular events, apart from the higher prevalence of traditional cardiovascular risk factors [2–4]. Current cardiovascular risk assessment tools using traditional cardiovascular risk factors may not reflect the actual risk of cardiovascular events in patients with TAK, and a disease-specific risk score is needed [4]. Although a previous study reported that in addition to traditional cardiovascular risk factors, anemia and low body mass index are factors independently associated with increased cardiovascular events [14], data regarding TAK disease-specific factors associated with cardiovascular events are still limited.

In this study, we aimed to identify TAK disease-specific factors associated with the risk of cardiovascular events, independent of the traditional cardiovascular risk factors.

## Methods

### Patients

In this retrospective cohort study, patients diagnosed with TAK and followed between 2006 and 2019 at two tertiary referral hospitals were included. All patients fulfilled the American College of Rheumatology (ACR) 1990 criteria for the classification of TAK [15]. Patients who concurrently had other chronic inflammatory diseases such as spondyloarthritis and inflammatory bowel diseases were excluded. Patients with a history of cardiovascular events or chronic kidney disease prior to the diagnosis of TAK were also excluded. Electronic medical records of each patient were reviewed from the index date (i.e., the date of diagnosis of TAK) to the last follow-up date. Data regarding traditional cardiovascular risk factors, TAK disease-specific factors, and incident cardiovascular events in each patient were extracted.

This study was approved by the institutional review board (IRB) of Gangnam Severance Hospital (IRB No. 3-2019-0423). Owing to the retrospective nature of this study, the requirement for informed consent was waived.

### Covariates

Regarding traditional CV risk factors, age, sex, presence of hypertension, use of antihypertensive drugs, systolic and diastolic blood pressure (BP), presence of diabetes mellitus, smoking status (current smoker or not), and lipid profile (total cholesterol, triglycerides, high-density lipoprotein [HDL] cholesterol, and low-density lipoprotein [LDL] cholesterol) at index date were evaluated. The 2008 Framingham 10-year risk score of general cardiovascular disease [16] was calculated based on collected data. Body mass index (BMI) and hemoglobin levels at index date were also evaluated. In regard to TAK disease-specific factors, erythrocyte sedimentation rate (ESR), C-reactive protein (CRP) levels, type of vascular involvement according to the Hata classification [17], involvement of pulmonary arteries, presence of aortic regurgitation, use of glucocorticoids, aspirin, and statins at index date were evaluated. The use of glucocorticoids was categorized as none-to-low and medium-to-high dose according to the dose received at the index date. A glucocorticoid dose of ≤ 7.5 mg prednisolone equivalent a day was considered none-to-low dose, and a glucocorticoid dose of > 7.5 mg and ≤ 100 mg prednisolone equivalent a day was considered medium-to-high dose [18]. The use of methotrexate (MTX) and azathioprine (AZA) until the last follow-up date or the date of incident cardiovascular event, whichever came first, was also assessed. The medication was considered “used” if it had been received for at least 3 months.

### Definition of cardiovascular events

Cardiovascular events were defined as documented episodes of coronary artery disease (angina, myocardial infarction, and revascularization procedures), cerebrovascular events (transient ischemic attack, and stroke; both cerebral infarction and hemorrhage), and congestive heart failure (both compensated and decompensated) that occurred between the index date and last follow-up date, as defined in a previous meta-analysis [19]. Shortness of breath or non-specific ST-T change in electrocardiogram or paroxysmal arrhythmia, respectively, was not considered as a cardiovascular event.

### Statistical analysis

To summarize patient characteristics, continuous variables were expressed as mean ± standard deviation or median (interquartile range) for parametric or non-parametric variables, respectively, while categorical variables were expressed as numbers (%). The normality of continuous variables was evaluated using the Kolmogorov–Smirnov test. Characteristics of patients who did and did not develop cardiovascular events were compared. Student’s *t* test or Mann–Whitney U test was performed for parametric or non-parametric continuous variables, respectively, and *χ*^2^ test (or Fisher’s exact test) was used to compare categorical variables. To estimate the risk of cardiovascular events according to potential TAK disease-specific factors, multivariable Cox proportional hazard regression analysis adjusted for traditional cardiovascular risk factors (age, sex, total cholesterol, HDL cholesterol, hypertension, diabetes mellitus, and smoking history) [16] was performed. The proportional hazards assumption was confirmed by examination of log (− log [survival]) curves and by testing Schoenfeld partial residuals; no relevant violations were found. For the interpretation of drug effects, adjustment with propensity score using inverse probability of treatment weighting (IPTW) was performed to minimize channeling bias. A *P* value of < 0.05 was considered statistically significant. All analyses were conducted using the SPSS software (version 25.0; IBM Corporation, Armonk, NY, USA).

## Results

### Incidence of cardiovascular events

A total of 207 patients with TAK who fulfilled the ACR 1990 classification criteria were included in the analysis. The median follow-up duration was 5.6 (2.1–10.9) years. Majority of the patients were female (174 of 207, 84.1%), and the mean age was 39.5 ± 13.2 years (Table 1). Cardiovascular events occurred in 41 (19.8%) patients, in median 3.0 (1.0–6.4) years of follow-up. Of the 41 cardiovascular events, 23 were coronary arterial events, 15 were cerebrovascular events, and 3 were congestive heart failure. The incidence of cardiovascular events was 41 per 1383.9 person-years.

**Table 1:** Characteristics of 207 patients with TAK

	*N* = 207
Age, years, mean (±SD)	39.5 ± 13.2
Female sex, *n* (%)	174 (84.1)
Hypertension, *n* (%)	100 (48.3)
Use of antihypertensive drugs, *n* (%)	100 (48.3)
Systolic BP, mmHg, mean (±SD)	129.3 ± 16.1
Diastolic BP, mmHg, mean (±SD)	75.6 ± 12.6
Diabetes mellitus, *n* (%)	19 (9.2)
Smoking, *n* (%)	18 (8.7)
Total cholesterol, mg/dl, mean (±SD)	180.8 ± 41.0
Triglycerides, mg/dl, mean (±SD)	112.6 ± 66.4
HDL cholesterol, mg/dl, mean (±SD)	56.1 ± 17.0
LDL cholesterol, mg/dl, mean (±SD)	102.2 ± 32.7
Framingham risk score, %, median (IQR)^a^	4.1 (2.5–8.0)
BMI, kg/m^2^, mean (±SD)	22.5 ± 3.1
Hemoglobin, g/dl, mean (± SD)	12.3 ± 1.5
Anemia, *n* (%)	84 (40.6)
ESR, mm/h, median (IQR)	40.0 (20.0–67.0)
CRP, mg/l, median (IQR)	4.0 (1.0–22.0)
Type of vascular involvement, *n* (%)	
I	48 (23.2)
IIA	23 (11.1)
IIB	36 (17.4)
III	8 (3.9)
IV	11 (5.3)
V	81 (39.1)
Pulmonary artery involvement, *n* (%)	16 (7.7)
Aortic regurgitation, *n* (%)	61 (29.5)
Use of glucocorticoids, *n* (%)	
None-to-low dose	85 (41.1)
Medium-to-high dose	122 (58.9)
Use of MTX, *n* (%)	126 (60.9)
Use of AZA, *n* (%)	38 (18.4)
Use of aspirin, *n* (%)	123 (59.4)
Use of statins, *n* (%)	123 (59.4)
Cardiovascular events, *n* (%)	41 (19.8)
Coronary arterial events	23 (11.1)
Cerebrovascular events	15 (7.2)
Congestive heart failure	3 (1.4)

*TAK* Takayasu arteritis, *BP* blood pressure, *HDL* high-density lipoprotein, *LDL* low-density lipoprotein, *BMI* body mass index, *ESR* erythrocyte sedimentation rate, *CRP* C-reactive protein, *MTX* methotrexate, *AZA* azathioprine, *SD* standard deviation, *IQR* interquartile range

^a^Patients aged < 30 years were excluded as per the formula of calculation

### Comparison between patients who did and did not develop cardiovascular events

Compared with patients who did not develop cardiovascular events, patients who developed cardiovascular events were older (38.5 ± 13.4 years vs. 43.6 ± 11.8 years, *p* = 0.028), more commonly had diabetes mellitus (6.6% vs. 19.5%, *p* = 0.029), had lower HDL cholesterol levels (57.3 ± 17.1 mg/dl vs. 51.2 ± 15.7 mg/dl, *p* = 0.040), more commonly exhibited a type V vascular involvement pattern (33.1% vs. 63.4%, *p* < 0.001) at the index date, and less commonly received MTX during the follow-up (65.1% vs. 43.9%, *p* = 0.013) (Table 2). There was no significant difference in sex distribution, prevalence of hypertension, use of antihypertensive drugs, systolic and diastolic BP, smoking status, total cholesterol, triglycerides, LDL cholesterol, BMI, hemoglobin, ESR, CRP, prevalence of pulmonary artery involvement and aortic regurgitation, and use of glucocorticoids, aspirin, and statins at the index date. Importantly, no significant difference in the Framingham risk score was observed between the two groups (3.9 [2.4–7.1]% vs. 5.5 [2.9–9.7]%, *p* = 0.119).

**Table 2:** Comparison of patient characteristics according to the development of cardiovascular events during follow-up

	CV events not occurred (*N* = 166)	CV events occurred (*N* = 41)	*P* value
Age, years, mean (±SD)	38.5 ± 13.4	43.6 ± 11.8	0.028
Female sex, *n* (%)	138 (83.1)	36 (87.8)	0.464
Hypertension, *n* (%)	75 (45.2)	25 (61.0)	0.070
Use of antihypertensive drugs, *n* (%)	75 (45.2)	25 (61.0)	0.070
Systolic BP, mmHg, mean (± SD)	128.9 ± 15.8	130.9 ± 17.0	0.477
Diastolic BP, mmHg, mean (± SD)	75.2 ± 12.5	76.9 ± 13.2	0.439
Diabetes mellitus, *n* (%)	11 (6.6)	8 (19.5)	0.029
Smoking, *n* (%)	12 (7.2)	6 (14.6)	0.210
Total cholesterol, mg/dl, mean (± SD)	182.9 ± 40.4	172.9 ± 42.6	0.167
Triglycerides, mg/dl, mean (± SD)	108.6 ± 57.8	127.6 ± 91.1	0.209
HDL cholesterol, mg/dl, mean (± SD)	57.3 ± 17.1	51.2 ± 15.7	0.040
LDL cholesterol, mg/dl, mean (± SD)	103.8 ± 32.4	96.2 ± 33.8	0.183
Framingham risk score, %, median (IQR)^a^	3.9 (2.4–7.1)	5.5 (2.9–9.7)	0.119
BMI, kg/m^2^, mean (± SD)	22.5 ± 3.3	22.7 ± 2.7	0.702
Hemoglobin, g/dl, mean (± SD)	12.3 ± 1.5	12.3 ± 1.3	0.980
Anemia, *n* (%)	66 (39.8)	18 (43.9)	0.629
ESR, mm/h, median (IQR)	39.0 (20.5–68.5)	41.0 (20.0–66.3)	0.717
CRP, mg/l, median (IQR)	4.1 (1.0–23.1)	3.6 (1.0–15.2)	0.908
Type of vascular involvement, *n* (%)			
I	42 (25.3)	6 (14.6)	0.147
IIA	19 (11.4)	4 (9.8)	> 0.999
IIB	32 (19.3)	4 (9.8)	0.150
III	7 (4.2)	1 (1.6)	> 0.999
IV	11 (6.6)	0 (0.0)	0.126
V	55 (33.1)	26 (63.4)	< 0.001
Pulmonary artery involvement, *n* (%)	13 (7.8)	3 (7.3)	> 0.999
Aortic regurgitation, *n* (%)	44 (26.5)	17 (41.5)	0.060
Use of glucocorticoids, *n* (%)			
None-to-low dose	66 (39.8)	19 (46.3)	0.443
Medium-to-high dose	100 (60.2)	22 (53.7)	
Use of MTX, *n* (%)	108 (65.1)	18 (43.9)	0.013
Use of AZA, *n* (%)	33 (19.9)	5 (12.2)	0.255
Use of aspirin, *n* (%)	94 (56.6)	29 (70.7)	0.100
Use of statins, *n* (%)	97 (58.4)	26 (63.4)	0.561

*CV* cardiovascular, *BP* blood pressure, *HDL* high-density lipoprotein, *LDL* low-density lipoprotein, *BMI* body mass index, *ESR* erythrocyte sedimentation rate, CRP C-reactive protein, *MTX* methotrexate, *AZA* azathioprine, *SD* standard deviation, *IQR* interquartile range

^a^Patients aged < 30 years were excluded as per the formula of calculation

### Estimation of risk of cardiovascular events according to TAK disease-specific factors

Table 3 shows the results of Cox proportional hazard regression analyses estimating the risk of cardiovascular events according to TAK disease-specific factors. In the univariable analysis, type V vascular involvement was associated with increased risk of cardiovascular events compared with other types (unadjusted hazard ratio [HR] 2.741, 95% confidence interval [CI] 1.443–5.205, *p* = 0.002), whereas the use of MTX was associated with reduced risk of cardiovascular events (unadjusted HR 0.474, 95% CI 0.253–0.887, *p* = 0.020). After adjusting for traditional cardiovascular risk factors, the observed associations persisted (for type V vascular involvement, adjusted HR 2.852, 95% CI 1.474–5.518, *p* = 0.002; for the use of MTX, adjusted HR 0.515, 95% CI 0.268–0.993, *p* = 0.047). Furthermore, adjustment with propensity score using IPTW confirmed that use of MTX was associated with reduced risk of cardiovascular events (IPTW-adjusted HR 0.401, 95% CI 0.168–0.957, *p* = 0.040).

**Table 3:** Risk of cardiovascular disease according to TAK disease-specific factors

	Univariable analysis		Multivariable analysis^a^	
	Unadjusted HR (95% CI)	*P* value	Adjusted HR (95% CI)	*P* value
BMI	1.031 (0.926–1.146)	0.579	0.905 (0.796–1.029)	0.129
Anemia	0.880 (0.468–1.654)	0.691	0.924 (0.464–1.838)	0.821
ESR	1.001 (0.991–1.011)	0.878	0.996 (0.985–1.008)	0.551
CRP	0.995 (0.986–1.005)	0.326	0.993 (0.982–1.003)	0.183
Vascular involvement type				
Types other than V	1.000 (reference)		1.000 (reference)	
Type V	2.741 (1.443–5.205)	0.002	2.852 (1.474–5.518)	0.002
Pulmonary artery involvement	1.011 (0.311–3.288)	0.985	0.717 (0.193–2.655)	0.618
Aortic regurgitation	1.341 (0.703–2.558)	0.373	1.024 (0.505–2.077)	0.947
Use of glucocorticoids				
None-to-low dose	1.000 (reference)		1.000 (reference)	
Medium-to-high dose	0.780 (0.414–1.471)	0.443	1.030 (0.523–2.030)	0.932
Use of MTX	0.474 (0.253–0.887)	0.020	0.515 (0.268–0.993)	0.047
Use of AZA	0.592 (0.231–1.513)	0.273	0.768 (0.278–2.122)	0.611
Use of aspirin	1.763 (0.880–3.532)	0.110	1.426 (0.695–2.925)	0.333
Use of statins	1.220 (0.640–2.327)	0.546	0.789 (0.391–1.592)	0.508

*TAK* Takayasu arteritis, *HR* hazard ratio, *CI* confidence interval, *BMI* body mass index, *ESR* erythrocyte sedimentation rate, *CRP* C-reactive protein, *MTX* methotrexate, *AZA* azathioprine

^a^Adjusted for traditional cardiovascular risk factors (age, sex, systolic BP, use of antihypertensive drugs, total cholesterol, HDL cholesterol, diabetes mellitus, and smoking status)

## Discussion

In this study, we observed that type V vascular involvement was associated with increased risk of cardiovascular events, while the use of MTX was associated with reduced risk of cardiovascular events in patients with TAK. To our knowledge, this is the first study to examine TAK disease-specific factors associated with cardiovascular events.

Type V vascular involvement, which is the most extensive pattern of vascular involvement [17], was associated with approximately three times higher risk (adjusted HR 2.852) of cardiovascular events than the other types. It is challenging to determine whether this association is attributable to arterial vasculitic involvement itself or to accelerated atherosclerosis due to chronic systemic inflammation. Considering that ESR and CRP were not associated with the risk of cardiovascular events in the multivariable Cox proportional hazard regression analysis, we presume that the increased risk of cardiovascular events is more likely attributable to arterial vasculitic involvement than to the accelerated atherosclerosis by chronic inflammation. Indeed, ESR (37.0 [20.3–62.3] mm/h vs. 42.0 [20.0–69.0] mm/h, *p* = 0.612) and CRP (5.0 [1.0–23.0] mg/l vs. 3.1 [1.0–20.9] mg/l, *p* = 0.523) levels did not differ between patients with type V and those with other types of vascular involvement (data not shown in results). Interestingly, a previous study reported that small retinal vessel involvement in TAK may be associated with type V vascular involvement [20]. Similar to previous findings, we also found that type V vascular involvement may be associated with coronary artery or cerebral artery involvement, leading to increased risk of cardiovascular events. Moreover, we have previously observed that type V was the most common (4 of 7, 57.1%) type of vascular involvement in mortality cases caused by cardiovascular events, supporting type V vascular involvement as a risk factor of cardiovascular events [21].

MTX, which is commonly administered in patients with TAK to achieve remission or to spare glucocorticoid, is the cornerstone of RA treatment [22]. The beneficial effect of MTX on cardiovascular events has been reported in patients with RA: MTX was associated with 28–66% reduction in cardiovascular events [23–25]. Similar to the observations in patients with RA, we also found that the use of MTX was associated with approximately 60% reduced risk (IPTW-adjusted HR 0.401) of cardiovascular events in patients with TAK. Given the established association between chronic inflammation and atherosclerosis [11–13], the association between the use of MTX and reduced risk of cardiovascular events might be contributed by reduced disease activity (i.e., inflammation). However, the use of AZA, another medication effectively used for controlling the disease activity of TAK [26], was not associated with reduced risk of cardiovascular events in this study. In addition to the indirect effects mediated by reduction in disease activity, MTX exerts direct cardioprotective properties by promoting cholesterol efflux, improving endothelial function and reducing oxidative stress [27–30]. These cardioprotective effects might explain why MTX, but not AZA, was associated with reduced risk of cardiovascular events in our study.

A previous study reported that the use of aspirin reduced the risk of acute ischemic events, while the use of statins was not associated with the risk of cardiovascular events in patients with TAK [31]. Similarly, we found no significant association between the use of statins and risk of cardiovascular events in this study. However, we also did not observe a significant association between the use of aspirin and risk of cardiovascular events. The effect of aspirin seems controversial, with another study reporting no significant effect of aspirin on the risk of cardiovascular events [4], consistent with our present study. Aspirin and statins are commonly used for cardioprotection; therefore, their effects are likely to be confounded by indication. Prospective studies are needed to clearly assess the cardioprotective effect of these medications in patients with TAK.

Contrary to a report supporting the association of anemia and low BMI with increased cardiovascular events in patients with TAK [14], another study refuted such an association [4]. In the present study, we also did not find a significant association between these factors and cardiovascular events. The significance of anemia and low BMI as risk factors for cardiovascular events in patients with TAK remains unclear.

Interestingly, the Framingham risk score at the index date did not differ between patients who developed cardiovascular events and those who did not. This suggests that assessment of cardiovascular risk using the current assessment tools is not as accurate in patients with TAK as it is in the general population. Additional consideration of disease-specific factors, such as type V vascular involvement and the use of MTX as identified in this study, may lead to better cardiovascular risk assessment in patients with TAK.

Our study had several limitations. First, this was a retrospective study. Although we found some significant associations between TAK disease-specific factors and cardiovascular events, the causal relationship could not be fully addressed. Second, CRP levels were relatively low in the total population compared with other studies. This may have led to an underestimation of the association between CRP levels and cardiovascular events.

## Conclusion

In conclusion, we showed that type V vascular involvement was associated with increased risk of cardiovascular events, whereas the use of MTX was associated with reduced risk of cardiovascular events, independent of traditional cardiovascular risk factors. In addition to the traditional cardiovascular risk factors, these TAK disease-specific factors may aid in the estimation of a more accurate disease-specific risk score for cardiovascular events in patients with TAK.

## Data Availability

All data generated or analyzed during this study are included in this article.

## Abbreviations

TAK: Takayasu arteritis
RA: Rheumatoid arthritis
ACR: American College of Rheumatology
BP: Blood pressure
HDL: High-density lipoprotein
LDL: Low-density lipoprotein
BMI: Body mass index
ESR: Erythrocyte sedimentation rate
CRP: C-reactive protein
MTX: Methotrexate
AZA: Azathioprine
IPTW: Inverse probability of treatment weighting
HR: Hazard ratio
CI: Confidence interval
